# Association of mandibular functional impairment and orofacial pain with subtypes of male sexual dysfunction: a cross-sectional study

**DOI:** 10.1590/1516-3180.2026.3624.20072026

**Published:** 2026-08-31

**Authors:** Ümit Erkut, Abdurrahim Yildiz, Rüstem Mustafaoglu

**Affiliations:** I Asst. Professor, Physiotherapy and Rehabilitation, Rumeli University, Istanbul, Türkiye.; II Assoc. Professor, Physiotherapy and Rehabilitation, Sakarya Applied Science University, Sakarya, Türkiye.; III Assoc. Professor, Physiotherapy and Rehabilitation, Istanbul University-Cerrahpaşa, Istanbul, Türkiye.

**Keywords:** Delayed Ejaculation, Erectile Dysfunction, Orofacial Pain, Premature Ejaculations, Temporomandibular Joint Disorders, Male Sexual Dysfunction, Mandibular Function, Temporomandibular Joint, Temporomandibular Joint Dysfunction, Pain, Sexual Health

## Abstract

**BACKGROUND:**

Temporomandibular disorder (TMD) are among the most common causes of orofacial pain and have been associated with several systemic conditions. However, their relationship with male sexual dysfunctions (MSDs) remains largely unexplored.

**OBJECTIVES:**

To investigate the association between TMD symptoms and different subtypes of MSDs.

**METHODS:**

A cross-sectional study was conducted with 91 men aged 18 to 50, classified as having premature ejaculation (n = 33), delayed ejaculation (n = 10), or erectile dysfunction (n = 22), or being control participants (n = 26). TMD symptoms were assessed using the Mandibular Function Impairment Questionnaire (MFIQ), and sexual function was evaluated using the International Index of Erectile Function (IIEF), Premature Ejaculation Diagnostic Tool (PEDT), and intravaginal ejaculatory latency time (IELT).

**RESULTS:**

Significant group differences were found in the IELT and all IIEF subdomain (p < 0.001) values. Participants with delayed ejaculation had the longest IELTs, whereas those with erectile dysfunction had the shortest. PEDT scores were significantly higher in the premature-ejaculation and erectile-dysfunction groups than in the control group, with no significant difference observed between the delayed-ejaculation and control groups. Orofacial pain (cheek–temple–jaw) was more frequent in all MSD groups (p = 0.001), whereas temporomandibular joint pain (ear area) was particularly more prevalent in the delayed-ejaculation group. MFIQ scores indicated greater mandibular impairment primarily in the erectile-dysfunction group. Multinomial regression analysis results showed that orofacial pain independently predicted all MSD subtypes, but temporomandibular joint pain was specifically associated with delayed ejaculation.

**CONCLUSION:**

The results indicate a potential association between TMD-related symptoms and MSDs, suggesting that orofacial pain and functional jaw impairment may be related to male sexual health. These findings highlight the potential importance of using a multidisciplinary approach involving collaboration between dentists, urologists, and physical therapists to improve both jaw and sexual health outcomes.

## INTRODUCTION

Orofacial pain includes conditions causing pain in the mouth, face, head, and neck.^1^ Temporomandibular disorder (TMD) is the most common cause of nondental orofacial pain.^2^ Nociceptive signals from the temporomandibular joint (TMJ), masticatory muscles, and facial tissues are transmitted via the trigeminal ganglion to brainstem nuclei and higher cortical centers for pain perception.^3^ Sensory inputs converge at the spinal and trigeminal dorsal horns, and these integrated signals may affect trigeminal motor function, thus explaining the link between TMD and chronic pain syndromes. Central pathways may enable communication between the TMJ and other body regions.^4^ For instance, patients with endometriosis may feel pain in both the pelvis and same-side TMJ,^5^ and TMD symptoms are more common in those with polycystic ovary syndrome.^6,7^ Bruxism and painful intercourse are also related, suggesting shared pain mechanisms between the TMJ and pelvic girdle.^8^

Despite these observations, the potential relationship between TMD and male sexual dysfunctions remains largely unexplored. This represents a significant omission in the existing literature, given that both conditions are highly prevalent and multifactorial, with central sensitization, psychosocial factors, and neuromuscular dysfunction playing a strong role in their development.

Investigating this relationship is clinically meaningful for several reasons. Firstly, it could shed light on shared neurobiological mechanisms, such as central sensitization and altered pain modulation, which could explain coexisting symptoms in different parts of the body. Secondly, identifying a potential association could improve clinical assessment by encouraging a more holistic approach rather than a region-specific one. Thirdly, it could have significant implications for treatment strategies by suggesting that interventions targeting one region (e.g. the TMJ or pelvic floor) could influence symptoms in another.

## OBJECTIVE

Individuals with TMD show distinct health and functional features compared to those with sexual dysfunctions such as premature ejaculation (PE), delayed ejaculation (DE), and erectile dysfunction (ED).^9^ Understanding these differences and their possible associations is crucial for developing targeted, interdisciplinary treatments. Therefore, in this study, we aimed to examine the relationship between TMD and sexual dysfunction, focusing on their interactions and how sexual dysfunction may influence TMD-related pain and function.

## METHODS

### Study design and population

This study was conducted at Kurbaa Education and Consultancy Center, where individuals seeking help for sexual dysfunctions were evaluated between May 2024 and March 2025. A total of 91 volunteers participated. The controlled setting ensured consistent data collection and reliable study procedures. Participants were classified into four groups: PE, DE, ED, and Control. Group allocation followed standardized diagnostic tools and objective criteria. The PE group included men with an intravaginal ejaculatory latency time (IELT) of ≤ 1 minute,^10^ a Premature Ejaculation Diagnostic Tool (PEDT) score of ≥ 11, and symptoms persisting for ≥ 6 months with self-reported distress.^11^ The DE group comprised individuals with an IELT of ≥ 25 minutes or difficulty and or absence of ejaculation in ≥ 75% of encounters that was not explained by organic causes such as selective serotonin reuptake inhibitor use, diabetes, or neurological disorders.^12^ The ED group included men with an International Index of Erectile Function (IIEF)-erectile function value of ≤ 16 and inability to maintain an erection sufficient for intercourse, excluding secondary causes such as cardiovascular or medication-related issues. All male sexual dysfunctions had to have persisted for ≥ 6 months. Control participants reported no sexual dysfunction, and had an IELT of ≥ 2 minutes, a PEDT score of ≤ 8, and an IIEF-Erectile Function value of ≥ 22.^11,13^ All participants reported regular sexual activity, of one or more times per week for 6 months. All participants provided written informed consent before their inclusion. The study was approved by the Ethics Committee of Sakarya University of Applied Sciences Rectorate, under protocol number E.123903, dated 24 April 2024. All procedures were conducted in accordance with the ethical standards outlined in the Declaration of Helsinki.

### Data collection and definition

The demographic characteristics and TMJ symptoms were systematically evaluated. The functional limitations of the TMJ were assessed using the Mandibular Function Impairment Questionnaire (MFIQ).^14,15^ Sexual function was evaluated using the IIEF (including erectile function, orgasmic function, sexual desire, intercourse satisfaction, and overall satisfaction), and premature ejaculation was assessed using the PEDT and IELT. In addition, participants underwent clinical examinations performed by a single clinician with 14 years of experience in TMD, using the Axis I diagnostic criteria for temporomandibular disorders DC/TMD.^16^ The examiner was formally trained and calibrated in the application of the DC/TMD protocol to ensure procedural consistency. Together, these tools provided a comprehensive overview of TMJ impairments and sexual function for analyzing their potential interrelationship.

### Mandibular Function Impairment Questionnaire

The MFIQ is a self-administered tool that is used to assess perceived difficulty in mandibular movements and functions. It has 17 items that are rated on a five-point Likert scale, with total scores ranging from 0 (no impairment) to 68 (severe dysfunction). The MFIQ is widely used in clinical and research settings for detecting patient-reported outcomes in TMD.^14,15^

### Intravaginal ejaculatory latency time

The IELT objectively measures premature ejaculation, defined as the time from vaginal penetration to ejaculation. A shorter IELT indicates impaired ejaculatory control, often linked to reduced sexual satisfaction and increased psychological distress.^17^

### Premature Ejaculation Diagnostic Tool

The PEDT is a validated five-item self-report questionnaire that is used to assess the presence and severity of PE. Items are scored from 0 to 4, with scores of ≤ 8 indicating no PE, those of 9 or 10 indicating probable PE, and those of ≥ 11 indicating PE.^11^

### The International Index of Erectile Function

The IIEF is a validated 15-item self-report questionnaire that is used to assess male sexual function, with higher values reflecting better function. The six-item erectile function domain is scored as follows: 22–25, no ED; 17–21, mild ED; 12–16, mild to moderate ED; 8–11, moderate ED; and 5–7, severe ED.^13^

### Delayed ejaculation

In the Diagnostic and Statistical Manual of Mental Disorders (5th ed.), DE is defined as a marked delay or absence of ejaculation in 75% to 100% of partnered sexual encounters, causing significant distress.^12^

### Statistical analysis

Statistical analyses were performed using the IBM SPSS Statistics for Windows version 27.0 (IBM Corp., Armonk, New York). Descriptive statistics are presented as mean ± standard deviation for continuous variables, whereas categorical variables are expressed as frequencies and percentages. Normality assumptions were assessed using the Shapiro–Wilk test. Given the robustness of using analysis of variance (ANOVA) to moderate deviations from normality, parametric analyses were considered appropriate. To assess differences among groups, one-way ANOVA was performed. When statistically significant differences were observed, post hoc pairwise comparisons were conducted using Bonferroni correction. Statistical significance was set at p < 0.05 for the initial ANOVA, and the Bonferroni-adjusted comparisons were evaluated accordingly to control for multiple testing. To explore the effect of pain localization, two separate multinomial regression models were constructed: one including orofacial (cheek–temple–jaw) pain and one including TMJ (ear area) pain, each adjusted for age. Orofacial pain was defined as self-reported pain in the cheek, temple, or jaw whereas TMJ pain referred specifically to pain localized around the ear area. An a priori power analysis was conducted using G*Power (version 3.1)^18^ to determine the minimum sample size required to detect a large effect size with adequate statistical power. Specifically, a one-way ANOVA test was planned, assuming an alpha level of 0.05, a desired power of 0.8, and a large effect size (Cohen's f = 0.4) based on conventional guidelines. The results of the analysis indicated that a total sample of 76 participants would be sufficient to detect statistically significant group differences.

## RESULTS

A total of 91 participants were included across the four groups: PE (n = 33), DE (n = 10), ED (n = 22), and Control (n = 26). The mean age differed among the groups (F = 3.21; p = 0.027), with participants in the DE group being the youngest (29 ± 5.65 years). However, this difference did not remain statistically significant after Bonferroni correction (p < 0.0167). There were no statistically significant differences in terms of height, weight, or body mass index among the groups (p > 0.05). The IELT differed significantly across groups (p < 0.001). Post hoc Bonferroni analysis showed that the DE group had a significantly longer IELT (4,980.00 ± 2,562.03 seconds) than the PE (50.15 ± 19.78 seconds; p < 0.001), ED (190 ± 103.18 seconds; p < 0.001), and Control (796.15 ± 392.36 seconds; p < 0.001) groups; all remained significant after Bonferroni correction. No significant differences were found among the groups in terms of marital status or education level (p = 0.184 and p = 0.177, respectively; Table 1).

**Table 1:** Characteristics of the participants

Characteristic	PE group (n = 33)	DE group (n = 10)	ED group (n = 22)	Control group (n = 26)	p Value
	Mean ± SD or n (%)	Mean ± SD or n (%)	Mean ± SD or n (%)	Mean ± SD or n (%)	
Age, years	**33.12** ± **7.25**	**29**±**5.65**	**36.31**±**7.23**	**32.07**± **4.83**	**0.027** a
Height, cm	178.63 ± 6.19	178.8 ± 5.45	178.18 ± 4.69	177.69 ± 6.13	0.922^a^
Weight, kg	83.54 ± 12.06	77.8 ± 10.31	82.90 ± 10.95	77.13 ± 9.42	0.694^a^
BMI, kg/m²	26.18 ± 3.59	24.3 ± 2.75	26.17 ± 3.77	26.11 ± 4.3	0.545^a^
IELT, seconds	50.15 ± 19.78	4,980 ± 2,562.03	190 ± 103.18	796.15 ± 392.36	**<0.001** a
Marital status – Married	15 (45.5)	6 (60)	14 (63.6)	19 (73.1)	0.184^b^
Education–University	24 (72.7)	10 (100)	16 (72.7)	23 (88.5)	0.177^b^

PE, premature ejaculation; n, number; SD, standard deviation; DE, delayed ejaculation; ED, erectile dysfunction; BMI, body mass index; IELT, intravaginal ejaculation latency time.

a One-way analysis of variance; significance level set at p < 0.05.

b Chi-squared test; significance level set at p < 0.05.

The comparison of sexual function parameters revealed significant differences across all IIEF domains and PEDT scores (p < 0.001; Table 2). The results of the post hoc analysis indicated that the ED group had lower values than the Control group in the Erectile Function, Orgasmic Function, and Sexual Satisfaction domains of the IIEF (p < 0.001), all of which remained significant after Bonferroni correction. Lower IIEF-Sexual Satisfaction values were achieved in the PE, DE, and ED groups than in the Control group (p < 0.001). For IIEF-General Satisfaction, both the PE (p < 0.005) and ED groups (p < 0.001) achieved lower values than the Control group. The PEDT scores were higher in the PE and ED groups than in the Control group (p < 0.001), indicating greater ejaculatory dysfunction.

**Table 2:** Comparison of sexual function parameters between the groups

Parameter	PE group (n = 33)	DE group (n = 10)	ED group (n = 22)	Control group (n = 26)	F	p Value^a^	Bonferroni correction with groups^b^
	Mean ± SD	Mean ± SD	Mean ± SD	Mean ± SD			
IIEF-Erectile Function	25.27 ± 4.79	24.70 ± 4.54	12.90 ± 6.72	28.07 ± 1.52	46.76	0.001	PE–DE 1.00 PE–ED **0.001** PE–C 0.154 DE–ED **0.001** DE–C **0.001** ED–C **0.001**
IIEF-Orgasmic Function	8.21 ± 2.07	7.10 ± 1.72	4.18 ± 3.11	9.15 ± 1.15	23.89	0.001	PE–DE 0.929 PE–ED **0.001** PE–C 0.588 DE–ED **0.004** DE–C 0.71 ED–C **0.001**
IIEF-Sexual Desire	7.75 ± 1.58	7.10 ± 0.73	5.95 ± 1.49	8.61 ± 1.13	15.67	0.001	PE–DE 1.00 PE–ED **0.001** PE–C 0.116 DE–ED 0.189 DE–C 0.023 ED–C **0.001**
IIEF-Sexual Satisfaction	9.81 ± 2.57	8.60 ± 4.22	5.63 ± 3.57	12.88 ± 1.36	26.82	0.001	PE–DE 1.00 PE–ED **0.001** PE–C **0.001** DE–ED 0.042 DE–C **0.001** ED–C **0.001**
IIEF-General Satisfaction	7.39 ± 2.06	7.30 ± 0.94	4.72 ± 1.98	8.92 ± 0.97	24.68	0.001	PE–DE 1.00 PE–ED **0.001** PE–C **0.005** DE–ED **0.001** DE–C 0.071 ED–C **0.001**
PEDT	12.93 ± 3.15	4.30 ± 2.26	9.04 ± 3.77	4.65 ± 2.07	45.98	0.001	PE–DE **0.001** PE–ED **0.001** PE–C **0.001** DE–ED **0.001** DE–C 1.00 ED–C **0.001**

PE, premature ejaculation; n, number; SD, standard deviation; DE, delayed ejaculation; ED, erectile dysfunction; C, control; IIEF, International Index of Erectile Function; PEDT, Premature Ejaculation Diagnostic Tool.

a One-way analysis of variance; significance level set at p < 0.05.

b One-way analysis of variance followed by Bonferroni post hoc correction; significance level set at p < 0.05.

The comparison of temporomandibular symptom parameters showed differences between groups (Table 3). Pain localized around the region of the ears was more frequently reported in the PE (45.5%), DE (80%), and ED (36.4%) groups than in the Control group (23.1%; p = 0.017). Similarly, pain in the cheeks, temples, or jaw was more common in the PE (69.7%), DE (80%), and ED (63.6%) groups than in the Control group (23.1%; p = 0.001), and this difference remained after Bonferroni correction. Regarding functional impairment, significant differences were observed in MFIQ scores among the groups. The MFIQ-Total scores differed significantly at the ANOVA level (F = 10.72; p < 0.001). The results of the post hoc analysis demonstrated that these scores differed significantly between the ED and Control groups (p < 0.001). However, the difference between the DE and Control groups (p = 0.0028) also remained significant after Bonferroni correction. Comparisons that did not meet the adjusted threshold (p < 0.0167) were considered not statistically significant. In terms of subdomains, the Control group achieved lower scores in the MFIQ-Function (F = 7.22; p < 0.001) than the PE (p = 0.018), DE (p = 0.003), and ED (p < 0.001) groups. After Bonferroni correction, the differences remained significant for the DE and ED groups, whereas the PE group did not meet the adjusted significance threshold. Similarly, the Control group had lower scores in the MFIQ-Nutrition subdomain (F = 12.53; p < 0.001) than the DE (p = 0.003) and ED (p < 0.001) groups; both remained significant after Bonferroni correction.

**Table 3:** Comparison of temporomandibular symptom parameters between the groups

Symptom		PE group (n = 33)	DE group (n = 10)	ED group (n = 22)	Control group (n = 26)	F	p Value	Bonferroni correction with groups^c^
		Mean ± SD or n (%)	Mean ± SD or n (%)	Mean ± SD or n (%)	Mean ± SD or n (%)			
Have you ever felt pain in the joint around your ears?	Yes/No	15 (45.5)/18 (54.5)	8 (80)/2 (20)	8 (36.4)/14 (63.6)	6 (23.1)/20 (76.9)		0.017^a^	
Have you ever felt pain around your cheeks, temples or jaw?	Yes/No	23 (69.7)/10 (30.3)	8 (80)/2 (20)	14 (63.6)/8 (36.4)	6 (23.1)/20 (76.9)		<0.001^a^	
Have you ever heard a noise when opening or closing your jaw?	Yes/No	22 (66.7)/11 (33.3)	6 (60)/4 (40)	8 (57.1)/6 (42.9)	9 (34.6)/17 (65.4)		0.093^a^	
Have you ever had difficulty opening your mouth to the point where two fingers can fit?	Yes/No	4 (12.1)/29 (87.9)	0 (0)/10 (100)	2 (9.1)/20 (90.9)	2 (7.6)/24 (92.4)		0.057^a^	
Do you have any parafunctional habits?	Yes/No	17 (51.5)/16 (48.5)	6 (60)/4 (40)	8 (36.4)/14 (63.6)	6 (23.1)/20 (76.9)		0.203^a^	
Degree of mouth opening		44.27 ± 3.69	43.30 ± 5.49	44.63 ± 5.14	45.03 ± 3.48	0.44	0.722^b^	
MFIQ-Function		2.45 ± 2.77	3.91 ± 3.21	4.04 ± 3.06	0.76 ± 1.63	7.22	<0.001^b^	PE–DE 0.791 PE–ED 0.184 PE–C 0.100 DE–ED 1 DE–C **0.012** ED–C **0.001**
MFIQ-Nutrition		1.75 ± 2.03	2.80 ± 1.93	3.5 ± 2.63	0.23 ± 0.51	12.53	<0.001^b^	PE–DE 0.806 PE–ED **0.008** PE–C **0.018** DE–ED 1 DE–C **0.003** ED–C **0.001**
MFIQ-Total		4.21 ± 4.50	6.70 ± 4.80	7.54 ± 5.27	10 ± 2.05	10.72	<0.001^b^	PE–DE 0.635 PE–ED 0.031 PE–C **0.028** DE–ED 0.635 DE–C **0.003** ED–C **0.001**

PE, premature ejaculation; n, number; SD, standard deviation; DE, delayed ejaculation; ED, erectile dysfunction; MFIQ, Mandibular Function Impairment Questionnaire; C, control.

a Chi-squared test; significance level set at p < 0.05. Participants in the control group reported significantly less pain in the joint areas around the ears, cheeks, temples, or jaw compared to the PE, DE, and ED groups.

b One-way analysis of variance; significance level set at p < 0.05.

c One-way analysis of variance followed by Bonferroni post hoc correction; significance level set at p < 0.05.

### Multinomial logistic regression analysis

In the primary multinomial regression model including orofacial (cheek–temple–jaw) pain and age, orofacial pain was significantly associated with PE, DE, and ED, but age was independently associated with ED only (Table 4A). In a secondary model, in which orofacial pain was replaced with TMJ (ear area) pain, TMJ pain was significantly associated with DE but not with ED, suggesting that pain localization may be related to different sexual dysfunction subtypes (Table 4B).

**Table 4A:** Multinomial logistic regression analysis including orofacial (cheek–temple–jaw) pain and age

Outcome (vs. Control group)	Predictor	OR	95% CI	p Value
PE	Age	1.03	0.94–1.13	0.492
	Orofacial pain	7.78	2.39–25.35	0.0007
DE	Age	0.91	0.79–1.06	0.228
	Orofacia pain	12.95	2.12–79.27	0.0056
ED	Age	1.1	1–1.21	0.041
	Orofacial pain	6.11	1.66–22.44	0.0064

OR, odds ratio; CI, confidence interval; PE, premature ejaculation; DE, delayed ejaculation, ED, erectile dysfunction. Reference group: control group (group 4). Orofacial pain was coded as 0 = absent, 1 = present.

**Table 4B:** Multinomial logistic regression analysis including temporomandibular joint (ear area) pain and age

Outcome (vs. Control group)	Predictor	OR	95% CI	p Value
PE	Age	1.01	0.93–1.1	0.809
	TMJ pain	2.7	0.84–8.65	0.095
DE	Age	0.86	0.73–1.01	0.07
	TMJ pain	12.99	1.30–129.91	0.002
ED	Age	1.09	1–1.19	0.058
	TMJ pain	1.52	0.41–5.61	0.527

OR, odds ratio; CI, confidence interval; PE, premature ejaculation; TMJ, temporomandibular joint; DE, delayed ejaculation, ED, erectile dysfunction. Reference group, normal individuals (group 4). TMJ pain coded as 0 = absent, 1 = present.

## DISCUSSION

We investigated the association between TMD and male sexual dysfunctions, including PE, DE, and ED, in this study. The findings indicate that, compared to healthy control participants, symptoms of TMD and functional impairments of the mandible were more prevalent in individuals with sexual dysfunction. These findings suggest a potential association between TMD-related pain and male sexual dysfunction. The results of the regression analyses indicated differential associations according to pain localization. Although generalized orofacial (cheek–temple–jaw) pain was statistically associated with all subtypes of sexual dysfunction (PE, DE, and ED), TMJ (ear area) pain was only significantly associated with DE. However, given the relatively small size of the DE subgroup, this finding should be interpreted with caution. Overall, these results may indicate that broader trigeminal nociceptive input could be related to sexual function parameters, although causal inferences cannot be drawn from the present cross-sectional design. In addition to pain-related factors, age was also independently associated with ED, underscoring the multifactorial nature of ED in this population. However, these findings should be interpreted with caution because unmeasured psychological, metabolic, and lifestyle-related factors may have influenced the observed associations.

Chronic pain, musculoskeletal tension, and limitations of movement commonly associated with TMD may be associated with activation of the sympathetic nervous system and disrupt autonomic balance, thereby affecting ejaculatory and erectile processes.^11,17^ Holstege^19^ highlighted the emotional motor system's regulatory control over pelvic organs, suggesting that trigeminal input may indirectly affect pelvic functions through brainstem and limbic system interactions.

The findings in the DE group are noteworthy. The results of the multinomial regression analysis indicated a potential association between TMJ (ear area) pain and DE (OR = 12.99). However, the wide confidence interval (95% CI: 1.30–129.91) indicates limited statistical precision, likely related to the small size of the DE subgroup (n = 10). Therefore, this result should be interpreted cautiously and considered preliminary until confirmed through studies involving larger cohorts.

DE is considered the least prevalent form of male sexual dysfunction, with epidemiological studies reporting rates of between 1% and 5%.^20^ This rarity may partly explain the limited number of participants with this dysfunction in our cohort. Nevertheless, the small subgroup size may have affected the stability of the regression estimates. Mechanistically, the coexistence of TMD symptoms may reflect shared neuromuscular and central sensitization processes. Trigeminal nociceptive input has been associated with altered somatosensory processing and autonomic modulation, which may influence pelvic motor control and ejaculatory function.^21,22^ This neurophysiological framework may help explain the association observed between orofacial pain and sexual dysfunction in the present study. Central sensitization in TMD, with reduced pain thresholds and enhanced somatosensory responsiveness, has been well documented.^22^ Moreover, trigeminal–spinal interactions may affect musculoskeletal regulation beyond the orofacial region, potentially extending to pelvic structures.^4^ Nevertheless, given the cross-sectional design and limited subgroup size, causal inferences cannot be drawn.

Dysregulation of the autonomic nervous system is implicated in both TMD and sexual dysfunction. Impaired baroreflex sensitivity, reduced heart rate variability, and elevated resting heart rate in patients with TMD were reported in the OPPERA study, suggesting persistent sympathetic overactivity.^23^ Because the autonomic nervous system plays a key role in erection and ejaculation, such imbalance may worsen sexual dysfunction. These findings align with prior research linking TMD-related pain, motor dysfunction, and autonomic dysregulation to reduced sexual performance, indicating that TMD may be associated with alterations in multiple physiological systems relevant to sexual health. Although psychological factors were not directly measured in this study, evidence in the literature indicates that stress and anxiety play a central role in the mechanisms of both TMD and male sexual dysfunctions. Future studies investigating the psychological dimensions of this relationship would significantly contribute to understanding the underlying causal links.

The findings emphasize the need for a multidisciplinary approach in managing men with coexisting TMD and sexual dysfunction. Clinical assessment should include both sexual history and TMD symptoms, with collaboration required among dentists, physiotherapists, urologists, and pain specialists to develop individualized treatment plans. Physical therapy modalities—such as transcutaneous electrical nerve stimulation, manual therapy, and myofascial release—may relieve pain and improve mandibular function. When combined with pelvic floor exercises, they can address overlapping dysfunctions. Techniques promoting autonomic balance, including heart rate variability-based biofeedback, diaphragmatic breathing, and craniosacral therapy, may reduce sympathetic overactivity and enhance orofacial and pelvic function.^15,24^ Pharmacological options such as gabapentinoids or centrally acting muscle relaxants may help decrease neural excitability and muscle tension, but should be used cautiously as adjuncts to conservative care.^25^

Recent evidence highlights a strong link between chronic musculoskeletal pain and sexual dysfunction, particularly in men. Katz et al.^26^ reported that individuals with chronic musculoskeletal pain frequently experience reduced sexual desire, satisfaction, and performance, with pain-related limitations, fatigue, and sympathetic overactivity interfering with sexual activity. Similarly, Widyadharma et al.^25^ identified shared mechanisms between chronic pain and erectile dysfunction, including hypothalamic-pituitary-adrenal-axis dysregulation, elevated proinflammatory cytokines, endothelial dysfunction, and autonomic imbalance. These factors both worsen pain and impair the vascular and neural components essential for sexual function. Collectively, these findings suggest that chronic pain—such as that associated with TMD—may contribute to sexual dysfunction through neurophysiological and psychophysiological mechanisms, confirming the need for multidisciplinary evaluation.

First, the cross-sectional design of this study limits causal inference. Second, the small sample size of the DE group (n = 10) is an important limitation. Although a statistically significant association with TMJ pain was observed, the wide confidence interval (95% CI: 1.30–129.91) indicates considerable uncertainty in the estimate. Therefore, findings related to the DE group should be interpreted as preliminary. Future studies with larger samples are needed to confirm these results and render them generalizable. Third levels of anxiety, depression, and stress—which are known to substantially influence both TMD and sexual functions—were not evaluated using standardized scales. Finally, relevant physiological and lifestyle factors, including metabolic status, hormonal profile, and overall physical activity level, were not comprehensively evaluated. The absence of these variables in the analysis prevents the exclusion of potential residual confounding. Furthermore, the statistically significant difference in mean age between groups may represent an additional confounding factor, because age is a well-established determinant of both sexual function and musculoskeletal health.

## CONCLUSION

The present study indicates a potential association between TMD and male sexual dysfunctions, suggesting that orofacial pain and functional jaw impairment may be related to male sexual health. Nevertheless, these findings remain preliminary and require confirmation through future studies with larger cohorts. The results highlight the importance of interdisciplinary collaboration among dentists, urologists, and physical therapists to support a more comprehensive and holistic management approach for affected patients. Addressing both orofacial and sexual dysfunctions may facilitate improved management of the complex interactions between chronic pain and autonomic dysregulation, ultimately contributing to enhanced quality of life in men with these conditions.

## Data Availability

The datasets generated and analyzed during the current study are included in this published article.
